# Supplementary material for: Learning is a fundamental source of individuality
Source: eLife. 2026 Sep 30;15:RP111235. doi: 10.7554/eLife.111235 (PMC13626652; doi:10.7554/eLife.111235)
Supplement: Peer review history

# Editor report: eLife Assessment

**DOI:** 10.7554/eLife.111235.3.sa0

**Authors:** Marta Zlatic

**Affiliations:** MRC Laboratory of Molecular Biology United Kingdom

**Keywords:** Compelling, Fundamental

This is a **fundamental** study of individual variation and the contribution of learning to behavioural individuality. The experimental design of massively parallel behavioural phenotypes is outstanding and the conclusions are supported by a **compelling** and rigorous analysis across a large number of experiments in thousands of individuals across genotypes and conditions. The dataset further represents an advance in studying visual associative learning thanks to the ability to make longitudinal measurements of many behavioural decisions within the same animals. These results are a major contribution to the understanding of the sources of behavioural individuality.

# Referee report: Reviewer #1 (Public review)

**DOI:** 10.7554/eLife.111235.3.sa1

"Learning is a fundamental source of individuality," by Manna and colleagues, interrogates different sources of variation in individual behavior. The authors place individual flies in a Y-shaped arena, which is a common design in the field, and illuminate the arms of the Y with blue versus green light. They track the color preference of individual animals and also perform operant conditioning, meaning that they teach the fly to avoid a particular color/arm by generating a foot shock when the fly enters that arm. There are a number of things that are impressive about this setup: The authors are able to collect data on thousands of individual flies of many different strain backgrounds, and they demonstrate a strong change in color preference after conditioning. This is nice, because in past papers visual learning ability has been modest and difficult to study. To put a number on it, in this paper animals on average don't show a color preference at the start of the assay, spending around 30% of their time in the one arm illuminated green, and the remaining time in the two arms illuminated blue. After conditioning, the average animal spends only 23% of its time in the green arm.

The authors run 64 animals through the assay for each of 88 wild type strains (maybe? see Major Point 1 below) and see considerable strain-specific (genetic) variation in the change in time spent in the shocked color after conditioning. Some strains show no learning, while others spend <10% of their time in the shocked color after conditioning. They also, I believe, see that some strains have more variability across individuals, which would suggest that some strains have stronger canalization at the development or circuit function level than others-i.e. some genotypes produce more consistent copies of the individual, others less consistent copies. (Or, some genotypes produce robust circuits, and others produce noisy circuits.)

Finally, the authors argue statistically that learning itself increases variability in individual performance. This makes a lot of sense to me intuitively. Learning changes the physical/chemical properties of circuits in the brain, and because it evolves over time and interacts with environmental variables, it seems like it should send different animals down different channels. Or, at a conceptual level, if I learn to play the piano and my sister doesn't (because of some genetic difference between us or something stochastic), this learning experience will cause all sorts of other differences in our behavior as time passes. I also think the authors do have enough data to be able to make this finding. However, the presentation of the argument in this portion of the paper is hard for me to understand, and I am not an expert in statistics, so the strength of the result is difficult for me to evaluate.

Major points:

(1) It's difficult to track through the paper the number of animals tested for different assays. At the beginning, it says N=5632, which works out to 64 flies for each of the 88 DGRP strains. 64 happens to be the number of parallel Y arenas they have. Later in the methods, there's description of more variation within the set of 64 for each strain-two different parent sets per strain, different sexes, conditioned and un-conditioned. And, while the results text focuses on the color learning, the methods discuss additional assays (place learning, multi-day learning).

Given the numbers, does each run of the 64 mazes include all the tested flies of one strain, or are flies of many strains included in each batch? Do different flies do different assays (color, place, multi-day) or do they all do all the assays? Perhaps there is a table including this information already in the supplement, but I recommend making it much clearer in the main results text and methods. While the dataset is large, if it is split over many conditions and/or if batch and genotype confound each other, this will affect the robustness of the results and how strong the conclusions can be.

(2) The data presentation in Figure 1 is elegant and easy to follow, but getting into Figure 2 and subsequently, I get lost in the statistics and have trouble understanding what is being measured. My understanding of the big picture is that while genetics and individual randomness contribute a lot to behavior, the evidence for learning as an amplifier of individuality is that variance in behavior among animals of the same strain increases over time in the conditioned group (i.e. the group that is doing the most learning, or a specific kind of learning), but not in the control group. This idea is illustrated in the flattening distributions in the cartoons in Figure 1A. The authors should include graphs of the real data that use the same format as in that cartoon. Instead, the graphs present "residuals," and I don't know what those are. I suspect it's "variation left over after accounting for effects of strain and individual stochasticity." I see the residuals being tracked per strain over time in Figure 2H, but I don't see the change over time in other graphs. I'm looking for something simple like, "variation within the strain at the beginning of learning and at later time points in learning." (But I'm not sure exactly what instantaneous measurement would be the focus in longitudinal analyses of learning behavior.)

(3) Figure 3 is a cool stab at tracking down the precise mechanism by which stochastic environment interacts with learning to send individuals along different behavioral routes. But again, like in Figure 2, I don't have the sophisticated understanding of statistics to understand exactly what the graphs are telling me, or how they relate to the underlying measurements. I'm relying on the results text alone to reach a conceptual understanding and just taking the graphs on trust.

So, overall, the authors have a very nice body of work here and with the potential to add a new facet to our understanding of the origins of diversity in animal behavior. In addition to the interpretations they focus on here, this dataset also represents an advance in studying visual associative learning in general, and quite an amazing ability to make longitudinal measurements of many behavioral decisions within the same animals. Improving the data presentation to make it easier to follow for a larger swathe of researchers, especially in figures 2 and 3, will increase its potential impact.

Comment on revised version:

The authors have addressed my main points, including adding description of their statistical analyses and providing more detail about the different assays run and which animals were included in the same assay batches.

# Referee report: Reviewer #2 (Public review)

**DOI:** 10.7554/eLife.111235.3.sa2

Summary:

The authors set out to test the extent to which differences in learning capacity and experience contribute to behavioural variation in a genetically identical population under identical environmental conditions.

Strengths:

The authors developed and used a scaled-up version of a simple two-choice behavioural paradigm allowing them to test thousands of individuals across multiple genotypes. They then deployed clever and powerful statistical analysis methods and provided compelling evidence for a role of variability in learning in the expression of behavioural variation.

Weaknesses:

There are no major weaknesses, although some level of longitudinal analysis to strengthen the evidence for a strict definition of individuality would be a welcome extension of a future study. In addition, it would have been very interesting, although understandably beyond the current scope, to delineate a potential source of learning variability in the brain.

# Author comment: Author response

**DOI:** 10.7554/eLife.111235.3.sa3

**Authors:** Riddha Manna, Johanni Brea, Goncalo N Vasconcelos Braga, Alireza Modirshanechi, Ivan Tomić, Ana Marija Jaksic

**Affiliations:** École Polytechnique Fédérale de Lausanne Lausanne Switzerland; École Polytechnique Fédérale de Lausanne Lausanne Switzerland; École Polytechnique Fédérale de Lausanne Lausanne Switzerland; École Polytechnique Fédérale de Lausanne Lausanne Switzerland; École Polytechnique Fédérale de Lausanne Lausanne Switzerland; École Polytechnique Fédérale de Lausanne Lausanne Switzerland

The following is the authors’ response to the original reviews.

**Public Reviews:**

**Reviewer #1 (Public review):**

"Learning is a fundamental source of individuality," by Manna and colleagues, interrogates different sources of variation in individual behavior. The authors place individual flies in a Y-shaped arena, which is a common design in the field, and illuminate the arms of the Y with blue versus green light. They track the color preference of individual animals and also perform operant conditioning, meaning that they teach the fly to avoid a particular color/arm by generating a foot shock when the fly enters that arm. There are a number of things that are impressive about this setup: The authors are able to collect data on thousands of individual flies of many different strain backgrounds, and they demonstrate a strong change in color preference after conditioning. This is nice, because in past papers, visual learning ability has been modest and difficult to study. To put a number on it, in this paper, animals on average don't show a color preference at the start of the assay, spending around 30% of their time in the one arm illuminated green, and the remaining time in the two arms illuminated blue. After conditioning, the average animal spends only 23% of its time in the green arm.

The authors run 64 animals through the assay for each of 88 wild-type strains (maybe? see Major Point 1 below) and see considerable strain-specific (genetic) variation in the change in time spent in the shocked color after conditioning. Some strains show no learning, while others spend <10% of their time in the shocked color after conditioning. They also, I believe, see that some strains have more variability across individuals, which would suggest that some strains have stronger canalization at the development or circuit function level than others, i.e., some genotypes produce more consistent copies of the individual, others less consistent copies. (Or, some genotypes produce robust circuits, and others produce noisy circuits.)

Finally, the authors argue statistically that learning itself increases variability in individual performance. This makes a lot of sense to me intuitively. Learning changes the physical/chemical properties of circuits in the brain, and because it evolves over time and interacts with environmental variables, it seems like it should send different animals down different channels. Or, at a conceptual level, if I learn to play the piano and my sister doesn't (because of some genetic difference between us or something stochastic), this learning experience will cause all sorts of other differences in our behavior as time passes. I also think the authors do have enough data to be able to make this finding. However, the presentation of the argument in this portion of the paper is hard for me to understand, and I am not an expert in statistics, so the strength of the result is difficult for me to evaluate.

Major points

(1) It's difficult to track through the paper the number of animals tested for different assays. At the beginning, it says N=5632, which works out to 64 flies for each of the 88 DGRP strains. 64 happens to be the number of parallel Y arenas they have. Later in the methods, there's a description of more variation within the set of 64 for each strain, two different parent sets per strain, different sexes, conditioned and unconditioned. And, while the results text focuses on the color learning, the methods discuss additional assays (place learning, multi-day learning).

Given the numbers, does each run of the 64 mazes include all the tested flies of one strain, or are flies of many strains included in each batch? Do different flies do different assays (color, place, multi-day), or do they all do all the assays? Perhaps there is a table including this information already in the supplement, but I recommend making it much clearer in the main results text and methods. While the dataset is large, if it is split over many conditions and/or if batch and genotype confound each other, this will affect the robustness of the results and how strong the conclusions can be.

(2) The data presentation in Figure 1 is elegant and easy to follow, but getting into Figure 2 and subsequently, I get lost in the statistics and have trouble understanding what is being measured. My understanding of the big picture is that while genetics and individual randomness contribute a lot to behavior, the evidence for learning as an amplifier of individuality is that variance in behavior among animals of the same strain increases over time in the conditioned group (i.e., the group that is doing the most learning, or a specific kind of learning), but not in the control group. This idea is illustrated in the flattening distributions in the cartoons in Figure 1A. The authors should include graphs of the real data that use the same format as in that cartoon. Instead, the graphs present "residuals," and I don't know what those are. I suspect it's "variation left over after accounting for effects of strain and individual stochasticity." I see the residuals being tracked per strain over time in Figure 2H, but I don't see the change over time in other graphs. I'm looking for something simple like, "variation within the strain at the beginning of learning and at later time points in learning." (But I'm not sure exactly what instantaneous measurement would be the focus in longitudinal analyses of learning behavior.)

(3) Figure 3 is a cool stab at tracking down the precise mechanism by which a stochastic environment interacts with learning to send individuals along different behavioral routes. But again, like in Figure 2, I don't have the sophisticated understanding of statistics to understand exactly what the graphs are telling me, or how they relate to the underlying measurements. I'm relying on the results text alone to reach a conceptual understanding, and just taking the graphs on trust.

So, overall, the authors have a very nice body of work here, and with the potential to add a new facet to our understanding of the origins of diversity in animal behavior. In addition to the interpretations they focus on here, this dataset also represents an advance in studying visual associative learning in general, and quite an amazing ability to make longitudinal measurements of many behavioral decisions within the same animals. Improving the data presentation to make it easier to follow for a larger swathe of researchers, especially in figures 2 and 3, will increase its potential impact.

**Reviewer #2 (Public review):**

Summary:

The authors set out to test the extent to which differences in learning capacity and experience contribute to behavioural variation in a genetically identical population under identical environmental conditions.

Strengths:

The authors developed and used a scaled-up version of a simple two-choice behavioural paradigm, allowing them to test thousands of individuals across multiple genotypes. They then deployed clever and powerful statistical analysis methods and provided compelling evidence for a role of variability in learning in the expression of behavioural variation.

Weaknesses:

There are no major weaknesses, although some level of longitudinal analysis to strengthen the evidence for a strict definition of individuality would be a welcome extension of a future study. In addition, it would have been very interesting, although understandably beyond the current scope, to delineate a potential source of learning variability in the brain.

Following our provisional response to the reviewers, we have implemented these additions to the manuscript:

(1) We have added 7 additional tables (Table 1-6 and table 8) to the supplementary that detail how many individual flies were used in which of the seven separate experiments, how the individuals were distributed across genotypes, replicates and sexes, and how many were filtered out before the final analysis. At the bottom of each table, we added a short description of the type of experiment and a brief explanation of the filtering. The four smaller experiments where we tested the two mutant lines and one wild-type DGRP line were used primarily to test and validate the experimental platform and the behavioural paradigm used for the main experiment. In these four experiments we tested green place learning, blue place learning, green colour learning, blue colour learning in four separate batches of flies. In each of these experiments we used 192 individuals (64 individuals x 3 genotypes x 4 experiments = 768 individuals in total). In the multiday experiment we used 64 flies per genotype per each of the four groups of sequences of learning paradigms, in total 512 individual flies. Here, each of these 512 individuals were retested in different learning paradigms over 4 days (Table 5). The main experiment was the green place learning (Table 6) where we tested all 88 DGRP lines and again the two mutant lines was used to obtain the majority of the main results and conclusions (64 individuals x 90 genotypes = 5760 individuals). Lastly, additional 896 individuals were measured in the experiment using blue place learning paradigm to test the consistency of learning behaviour as opposed to colour bias within genotype (Table 8). In summary, in all experiments, we have always measured behaviour in 64 flies per genotype (full loading of the behavioural platform), and they were distributed almost entirely evenly across replicates, sexes, and conditions (control vs conditioned). No individual was reused across experiments. In most cases, after filtering the data, 60 or fewer individuals were used in final analyses. For the very few deviations from this experimental design (which occurred due to unforeseen events such as dropped/sick vials, flies flying away or accidentally squished during setup, skewed number of males and females etc.) we added a short explanation in the text below the tables. In total, across all reported experiments in this study, we measured behaviour in 7936 individuals.

(2) We have added a schematic visual representation of classical measurement of individuality (variance of the distribution of behaviour within genotype where genetically identical individuals are raised in the same environment), entropy-based measurement of individuality (residual individuality) and the change in residual individuality, as we use them in this study (Figure 2D). We also provide a list of different DH_resid_ measures and what distributions are being compared across the DH_resid_ in the same figure. We hope this will serve as a more intuitive explanation of individuality and help readers interpret and follow more easily the results that we report after this figure.

(3) In the same vein, we added another schematic visual representation to Figure 3 (Figure 3F) where we depict how distributions of individual behaviour may change with every decision and how this change translates to (or can be read out from) the change in residual individuality. We have also renamed the X axis of Figure 3E to “DH_resid_ Start”, so that it is clearer what is measured here and matches the explanation in Figure 2D.

(4) We have added two additional supplementary figures where the reader can inspect in more detail how the distributions of individual behaviour change longitudinally across time for each genotype in control and conditioned (Figure 3 – Figure supplement 2 and Figure 3 – Figure supplement 3). From these figures one can glance how variance as well as the shapes of the distributions change as the flies learn in the conditioned setting, and how they remain largely the same in the control where flies behave spontaneously. We have added a sentence in the main text to introduce these figures: “We found that the distributions of individual behaviour were broader and their shapes changed substantially over the course of the experiment for the conditioned flies, and not for the control flies Figure 3 - figure supplement 2, Figure 3 - figure supplement 3.”

(5) As noted in the first provisional response to reviewers, we changed the sentence “In every individual, behaviour is shaped by deterministic, genetic factors and by environmental events throughout lifetime, which may be stochastic and can occur at the molecular, cellular, organismal and even population scales.” to “In every individual, behaviour is shaped by fixed genetic factors and by variable environmental events throughout lifetime, which may be stochastic and can occur at the molecular, cellular, organismal and even population scales.”

(6) Some sentences were edited in the results so that we can correctly refer to the newly added tables and figures. Context, meaning or interpretation of the results in these sentences was not altered.

(7) While adding the new table references to the text, we noticed a typo that propagated in the previous version where the number of flies used in the main experiment was stated to be N = 5238, when in fact it should have been N=5239. This is now fixed.

We once again thank the reviewers for their comments and suggestions – we believe their suggestions helped us improve the presentation and interpretability of our study and we hope the reviewers and readers will agree with this as well.
